# Metabolic-Hormonal Interplay and the Case for a Reproductive-Metabolic Framework in the Glucagon-Like Peptide-1 (GLP-1) Era

**DOI:** 10.7759/cureus.111478

**Published:** 2026-06-25

**Authors:** Alisha Lakhani, Sahil Lakhani

**Affiliations:** 1 Medicine, Shantabaa Medical College, Amreli, IND; 2 Pharmacy, Parul University, Vadodara, IND

**Keywords:** contraception, glp-1 receptor agonists, hormonal contraception, hyperandrogenism, insulin resistance, metabolic-hormonal interplay, pcos, pmos, polycystic ovary syndrome, reproductive-metabolic axis

## Abstract

Polycystic ovary syndrome (PCOS), proposed by some investigators to be more accurately termed polycystic-morphology ovary syndrome (PMOS), reflecting its heterogeneous and not universally cystic pathophysiology, remains the most prevalent endocrine disorder among reproductive-age women worldwide. Despite this, its clinical management continues to be fragmented along specialty lines, with gynecologists addressing menstrual irregularity and contraception, endocrinologists managing insulin resistance and metabolic risk, and dermatologists treating hyperandrogenic manifestations.

This editorial proposes the Reproductive-Metabolic Axis (RMA) as a unifying conceptual framework for understanding PCOS/PMOS beyond its phenotypic surface. We argue that contraceptive decision-making in women with PCOS/PMOS cannot be separated from metabolic status, insulin resistance, androgen excess, and cardiovascular risk profiling. In the glucagon-like peptide-1 (GLP-1) receptor agonist era, which has transformed the management of obesity and insulin resistance, new clinical questions arise regarding the interaction between incretin-based therapies, hormonal contraception, and ovarian function restoration. The present editorial does not propose a validated therapeutic protocol, but rather a hypothesis-generating framework that invites further clinical investigation into whether integrated reproductive-metabolic management improves outcomes for women with PCOS/PMOS.

## Editorial

Polycystic ovary syndrome (PCOS) has been diagnosed, debated, and managed for decades, yet a clinical consensus on its fundamental nature remains elusive. The Rotterdam criteria of 2003, which defined PCOS through any two of the three features of oligo-anovulation, clinical or biochemical hyperandrogenism, and polycystic ovarian morphology on ultrasound, broadened the diagnostic umbrella substantially, capturing a heterogeneous population in which metabolic, reproductive, and dermatological manifestations coexist in highly variable combinations [1]. Recent discourse has questioned whether the term "polycystic" itself is clinically misleading: many patients meeting diagnostic criteria do not have cysts in the conventional pathological sense, and the ovarian morphology described by ultrasound reflects follicular arrest rather than true cyst formation. The proposal to rename the condition polycystic-morphology ovary syndrome (PMOS) reflects a growing recognition that nomenclature shapes clinical understanding, and that PCOS has historically been approached as a gynecological condition when its biology is fundamentally metabolic, endocrine, and systemic [2].

This framing matters enormously for contraceptive counseling. When a clinician encounters a woman with PCOS/PMOS seeking contraception, the conversation too often begins and ends with the menstrual calendar. Combined oral contraceptive pills (COCPs) are routinely offered as first-line management for cycle regulation and hyperandrogenic symptoms: hirsutism, acne, and seborrhea; and their contraceptive efficacy is beyond question [3]. However, the metabolic consequences of hormonal contraception in a population already predisposed to insulin resistance, dyslipidemia, low-grade inflammation, and cardiovascular risk are insufficiently integrated into routine clinical decision-making. Estrogen-containing contraceptives can impair insulin sensitivity and alter lipid profiles, effects that may be clinically meaningful in women with metabolically complex PCOS/PMOS phenotypes [3]. The choice between combined oral contraceptives, progestin-only pills, intrauterine devices, implants, or barrier methods is not merely a reproductive decision; it is a metabolic one. However, it must be acknowledged that the metabolic effects attributable to contraceptive choice are difficult to disentangle from those intrinsic to PCOS/PMOS itself, particularly in the presence of obesity, baseline insulin resistance, and phenotypic heterogeneity, all of which independently confound outcome attribution.

The complexity deepens when fertility is considered alongside contraception. Women with PCOS/PMOS frequently experience a paradox: they are anovulatory and may struggle to conceive, yet those who do ovulate unpredictably are at risk of unintended pregnancy during periods of partial cycle restoration, which can occur spontaneously, with lifestyle modification, or increasingly with pharmacologic metabolic intervention. The concept of contraceptive need in PCOS/PMOS is therefore not static. A woman who begins GLP-1 receptor agonist therapy for metabolic management may experience ovulation restoration during or after treatment, creating a reproductive window that is rarely discussed in obesity or metabolic medicine consultations [4]. Clinicians prescribing incretin-based therapies to women of reproductive age have an obligation to address contraceptive planning as part of metabolic treatment initiation; a conversation that does not currently occur systematically in most clinical pathways.

The GLP-1 receptor agonist era has transformed the landscape of metabolic medicine and, by extension, the clinical trajectory of PCOS/PMOS. Agents such as semaglutide and tirzepatide have demonstrated substantial improvements in body weight, insulin resistance, androgen levels, menstrual regularity, and markers of ovarian dysfunction in women with obesity-related PCOS/PMOS [4,5]. Semaglutide, in particular, has been associated with reductions in the free androgen index, improvements in menstrual cycle regularity, and restoration of ovulatory function in some clinical series, raising both therapeutic promise and reproductive safety considerations simultaneously. Weight reduction mediated by GLP-1 receptor agonists may partially restore the hypothalamic-pituitary-ovarian axis in women with obesity-driven anovulation, with implications for both fertility and unintended pregnancy risk that must be proactively addressed [4].

Yet metabolic improvement, as in other fields of medicine, does not guarantee reproductive normalization. Hormonal architecture in PCOS/PMOS is not purely driven by adiposity or insulin resistance. Genetic predispositions in steroidogenesis, gonadotropin secretion, and ovarian theca cell function contribute to hyperandrogenism and follicular arrest independently of metabolic status [1]. A woman who achieves meaningful weight reduction and insulin sensitization through GLP-1 receptor agonist therapy may still present with persistent hyperandrogenism, anovulatory cycles, and dermatological sequelae, with the Reproductive-Metabolic Axis (RMA) partially restored but not fully corrected. This creates the modern paradox of metabolic success and persistent reproductive-endocrine dysfunction, which mirrors the dermal challenge described in the post-GLP-1 body remodeling literature.

The RMA is proposed here as a conceptual framework for integrating these considerations into a unified clinical philosophy. The RMA does not define a validated treatment protocol. Rather, it repositions PCOS/PMOS as a condition at the intersection of metabolic biology and reproductive endocrinology, in which neither domain can be adequately managed in isolation. Contraceptive counseling in this framework becomes an individualized risk stratification exercise: assessing insulin resistance, lipid profile, cardiovascular risk, androgenic phenotype, menstrual pattern, weight trajectory, and planned fertility timeline before selecting a contraceptive method. The clinician must ask not only what will prevent pregnancy, but what will best serve the patient's metabolic and hormonal health across the reproductive lifespan.

Practical implications of the RMA framework are considerable. For women with metabolically complex PCOS/PMOS phenotypes, particularly those with insulin resistance, elevated fasting glucose, dyslipidemia, or early markers of cardiovascular risk, estrogen-containing contraceptives warrant careful metabolic review rather than reflexive prescription [3]. Progestin-only methods, levonorgestrel intrauterine systems, or etonogestrel implants may offer superior metabolic neutrality while providing cycle control and contraceptive efficacy. For women initiating GLP-1 receptor agonist therapy, contraceptive counseling should occur at the time of prescription initiation, with explicit discussion of ovulation restoration risk as metabolic improvement proceeds. For women with PCOS/PMOS in the peri-conceptional period, the interaction between incretin-based therapies and early pregnancy outcomes, including the potential teratogenic implications of GLP-1 receptor agonist exposure, requires careful counseling given that these agents are currently contraindicated in pregnancy [4,5].

Several important limitations of the RMA framework must be acknowledged. This editorial is conceptual and does not present original clinical data. PCOS/PMOS encompasses a broad and heterogeneous phenotypic spectrum, and recommendations derived from one subpopulation, for example, obese women with insulin-resistant PCOS, may not apply to lean phenotypes, adolescent presentations, or post-menopausal metabolic persistence. The evidence base for GLP-1 receptor agonists in PCOS/PMOS remains primarily derived from studies designed for obesity or type 2 diabetes management; dedicated reproductive endocrine trials in PCOS/PMOS populations are limited, and long-term data on ovulation restoration, fertility outcomes, and contraceptive interactions are scarce [4,5]. The renaming of PCOS as PMOS, while conceptually compelling, has not achieved consensus in international guidelines, and clinicians should continue to apply existing diagnostic criteria while remaining attentive to evolving nomenclature debates [2].

The central argument of this editorial is that PCOS/PMOS demands a clinical language that matches its biological complexity. Contraception is not a gynecological footnote in the management of this condition; it is a metabolic, hormonal, and reproductive decision with long-term implications for cardiovascular health, fertility planning, and quality of life. As GLP-1 receptor agonists become increasingly prescribed to women of reproductive age with PCOS/PMOS, the integration of reproductive counseling into metabolic medicine pathways is no longer optional. The RMA framework proposes that the future of PCOS/PMOS management lies in treating the whole biology, not the uterus, not the ovary, not the metabolic panel in isolation, but the woman whose health spans all three.
